# Transgender Health Care Curriculum Development: A Dual-Site Medical School Campus Pilot

**DOI:** 10.1089/heq.2019.0106

**Published:** 2020-04-02

**Authors:** Anna J. Najor, Juliana M. Kling, Reese L. Imhof, Jon D. Sussman, Todd B. Nippoldt, Caroline J. Davidge-Pitts

**Affiliations:** ^1^ Mayo Clinic Alix School of Medicine, Rochester, Minnesota.; ^2^ Division of Women's Health Internal Medicine, Mayo Clinic, Scottsdale, Arizona.; ^3^ Division of Endocrinology, Diabetes, Nutrition, Mayo Clinic, Rochester, Minnesota.

**Keywords:** curriculum development, implementation, health equity, transgender health care

## Abstract

**Purpose:** Lack of physician training contributes to health care disparities for transgender people. The limited generalizability and feasibility of published training approaches lessen their utility in lowering barriers for other institutions to adopt similar training.

**Methods:** All first-year medical students at the Mayo Clinic Alix School of Medicine (MCASOM) in Minnesota and Arizona received a 1-h lecture introducing key concepts related to transgender people and their health disparities. Students completed a 21-question survey before and after the lecture, and 1 year later. Chi-square likelihood coefficients were used to compare responses between the three time points.

**Results:** Eighty-six of 100 students answered the prelecture survey (86% response rate); 70 the postlecture survey; and 44 the 1-year follow-up survey. Twenty-five (29%) students had prior education in any lesbian, gay, bisexual, and transgender (LGBT+) health disparities, but this did not correlate with more favorable attitudes or knowledge. LGBT+ students and those with close LGBT+ friends had the most favorable attitudes and knowledge. The proportion of students comfortable with caring for transgender people changed significantly (76% self-reported very comfortable prelecture vs. 91% postlecture, *p*=0.0073) and remained at 89% 1 year later. The proportion of students comfortable with a transgender patient scenario significantly increased (67% self-reported very comfortable prelecture vs. 87% postlecture, *p*=0.032) even when surveyed 1 year later (95% very comfortable, *p*<0.0001).

**Conclusion:** This study demonstrates that a 1-h lecture can increase the proportion of medical students who demonstrate positive attitudes and correct knowledge on transgender patient care for at least a year, and how a survey can gather essential information on student learning needs to guide training development.

## Introduction

People who belong to gender and sexual orientation minority groups suffer worse health outcomes than the general population^1–3^ due to barriers in accessing medical care^4^ as well as discrimination^5^ that can lead to care refusal, inferior treatment, or even verbal abuse from health care workers.^6–9^ In addition to discrimination, lack of knowledge about special health care needs may exacerbate the quality deficit experienced by gender and sexual orientation minority groups. Transgender and gender-diverse individuals have gender identities that do not align with their sex recorded at birth.^4^ Many transgender individuals report having to educate their physicians on transgender-specific health care and cite lack of knowledgeable physicians as a major barrier to receiving quality care.^10–12^ In one survey study, as many as 40% of lesbian, gay, bisexual, and transgender (LGBT+) respondents report lack of physician training as a barrier to care.^6^ This is compounded by limited opportunities for health care practitioners to learn about caring for people of gender and sexual minorities and low self-efficacy for treating them.^13–18^ These deficits in knowledge contribute to health outcome disparities.^5,15^

To address these health outcome disparities, in 2007, the Association of American Medical Colleges (AAMC) advised medical schools to include curricula on knowledge, skills, and attitudes needed to best care for LGBT+ people, and outlined learning objectives and educational principles to support effective teaching.^16^ Medical school training on LGBT+ patient care cultivates an inclusive institutional climate and addresses discrimination toward patients since understanding more about minority groups positively impacts attitudes about them.^19^

Despite this, training is still insufficient. One survey of 2261 medical students found that over 53% felt inadequately prepared to address concerns related to sexuality.^20^ Similarly, another survey of 659 medical students at seven schools showed that over 50% of students felt their training and competency were lacking in treating people of gender minority status and those with differences in sex development.^21^ Only 5% of U.S. endocrinologists who responded to a knowledge survey had received training on caring for transgender people in medical school.^22^ A survey of deans at 176 U.S. and Canadian MD and DO schools found a median of 2 h of training on LGBT+ patient care during clinical years, a median of 5 h of training throughout all 4 years. Thirty-three percent of the deans reported that no time was dedicated to LGBT education, and when it did, 26% of deans perceived the training as “poor” or “very poor.”^14^

Publications on teaching methods on caring for gender and sexual minority people have increased in recent years. Multiple different curricular models have been evaluated, including 2-h seminars,^23^ standardized patient scenarios,^24^ as well as a multisession interdisciplinary LGBT patient care certificate program,^25^ all demonstrating various degrees of improvement in medical student knowledge and attitudes and varying degrees of adherence to prior literature and the AAMC guidelines. Regarding transgender-specific care, results of a longitudinal 10-h curriculum showed improve attitudes toward transgender people up to 3 months later,^26^ and even a single didactic session on the biology of gender resulted in a significant 67% improvement in student willingness to care for transgender people 1 month after the teaching.^27^

The Mayo Clinic Alix School of Medicine (MCASOM) is piloting a new curriculum to increase medical student competence and confidence in transgender and gender-diverse patient care. Students received an introductory lecture on transgender health information and disparities, and were surveyed before the lecture as well as immediately following and 1 year later. This study aims to identify the learning needs of the student body, assess the quality of the lecture, and evaluate the responses for attitude and knowledge at 1-year postlecture.

## Methods

### Lecture

A 1-h lecture was given to medical students in the fall of their first year at the MCASOM in 2017. Attendance was mandatory at both campuses, Rochester, MN, and Scottsdale, AZ. The lecture included an explanation of the spectrum of identities associated with gender expression and sexual orientation, a broad overview of LGBT+ health disparities, and the description of a patient scenario to demonstrate how subtle aggressions by medical staff may lead to less health care utilization and poorer treatment outcomes (Appendix Tables A1 and A2). Details of the lecture content between Rochester, MN, and Scottsdale, AZ, campuses varied slightly based on the expertise of the lecturers; however, the learning objective remained similar.

### Survey methodology

A 21-question voluntary, anonymous online survey focusing on transgender health was e-mailed to all 100 students pre- and postlecture using Mayo Clinic-licensed Research Electronic Data Capture (REDCap) survey platform (Appendix Table A3). Students were given a time frame of 1 week to complete the postlecture survey. An identical survey was distributed 1 year later to assess knowledge retention. The survey was developed using a previously validated survey^28^ as well as expert input from clinicians at the Mayo Clinic Transgender and Intersex Specialty Care Clinic for additional knowledge questions. Questions focused on comfort level with treating transgender patients and their personal beliefs and experiences with transgender people. Students' sociodemographic characteristics were also asked. Mayo Clinic Institutional Review Board found the survey exempt and MCASOM Student Protection Board approved the survey. Answers indicating more comfort with and acceptance of transgender patients are described as more “favorable” attitudes, whereas answers indicating more accurate beliefs regarding the nature of transgender people and their care needs are described as more “correct” answers and knowledge. Knowledge questions could be answered in yes/no or true/false format, whereas attitude questions could be answered with a four-level scale for comfort or agreement.

### Statistical methods

Chi-square likelihood coefficients were used to compare frequency of responses pre- and postlecture, and at 1-year follow-up. As the survey was anonymous, pre- and postlecture surveys could not be compared directly with a paired *t*-test. Alpha of 0.05 was used as the significance level. JMP Pro 13.0.0 (SAS Institute, Inc., Cary, NC) was used for data analysis.

## Results

### Prelecture survey

Eighty-six of 100 students completed the prelecture survey (86% response rate). Respondent characteristics are reported in Table 1. Some facts were known by a large proportion of students before the lecture. Eighty-four participants (98%) knew that not all transgender people have the same goals for transition. Eighty-four participants (98%) knew that it is important for students to know about their future patients' gender identity and sexual orientation. Eighty-five participants (99%) knew that transgender people have unique health risks and health needs. Eighty-three participants (97%) knew that sexual minorities have worse access to health care services.

**Table 1.** Respondent Characteristics

Question	n (%)
Gender	
Female	44 (51.2)
Male	42 (48.8)
Age	
20–24	64 (74.4)
25–29	16 (18.6)
30–34	5 (5.8)
35–39	1 (1.2)
Sexual orientation	
Straight/heterosexual	73 (85.9)
Gay/lesbian/homosexual	3 (3.5)
Bisexual	3 (3.5)
Prefer not to say	4 (4.7)
Prefer to self-describe	3 (Asexual, Fluid, Queer) (3.5)
Ethnicity	
Non-Hispanic or Latino or Spanish Origin	73 (84.9)
Hispanic or Latino or Spanish Origin	8 (9.3)
Prefer not to say	3 (3.5)
Missing	2 (2.3)
Race	
White	61 (70.9)
Asian	18 (20.9)
Black or African American	1 (1.2)
Prefer not to say	5 (5.8)
Missing	1 (1.2)
Religion	
Christian	34 (39.5)
Atheist	19 (22.1)
Hindu	5 (5.8)
Muslim	3 (3.5)
Jewish	2 (2.3)
Other	17 (19.8)
Prefer not to say	5 (5.8)
Missing	1 (1.2)
Environment you grew up in	
Suburban	56 (65.1)
Urban	19 (22.1)
Rural	10 (11.6)
Missing	1 (1.2)
How much does your religion (spirituality) impact you opinion on sexual practices, sexual orientation, family values, gender, and reproductive issues?	
Not at all	54 (62.8)
Somewhat	23 (26.7)
Extremely	7 (8.1)
Prefer not to say	2 (2.3)
Have you had close friends who are LGBT?	
Yes	59 (68.6)
No	27 (31.4)
Have you received education in LGBT disparities?	
Yes	25 (29.1)
No	61 (70.9)

LGBT, lesbian, gay, bisexual, and transgender.

**FIG. 1.**
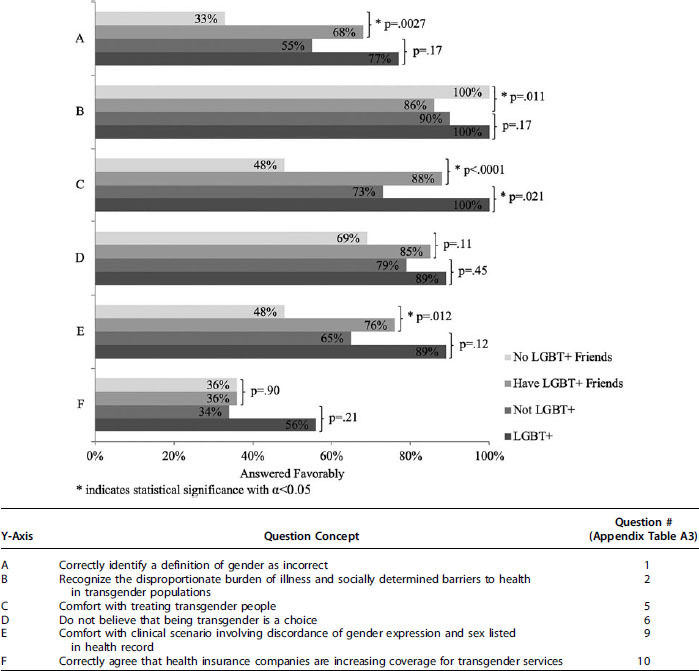
Prelecture proportion of favorable or correct responses and chi-square comparison between student groups.

Figure 1 depicts the differences in baseline knowledge and attitudes between students who have and have not had close LGBT+ friends and between students who are and are not LGBT+. Questions that nearly all students answered favorably, as listed previously, are not included in Figure 1. Having had close LGBT+ friends and being LGBT+ have the strongest correlation with answering attitude questions more favorably and knowledge questions correctly, compared with those without close LGBT+ friends and those who are not LGBT+.

Students who believe that being transgender is a choice are significantly more likely to be uncomfortable treating transgender patients (*p*=0.0026), and less comfortable with the gender minority patient scenario (*p*=0.0016), compared with those who do not believe being transgender is a choice. Students from urban environments are significantly more likely to be comfortable treating transgender people than students from suburban or rural environments (*p*=0.014). Students from suburban environments are significantly less likely to have had close friends who are LGBT+ (*p*=0.025) than students from urban or rural environments. Older students demonstrate more favorable attitudes and more knowledge compared with younger students, but this is only statistically significant in their ability to recognize the difference between gender and sexual orientation (*p*=0.0078). Students who report no influence of their religion on their opinions are more likely to correctly identify the difference between gender and sexual orientation (*p*=0.02), are more comfortable treating transgender people (*p*<0.0001), are more likely to report that being transgender is not a choice (*p*=0.0005), are more likely to be comfortable with the gender minority patient scenario (*p*=0.012), and are less likely to report that transgender people have unique disease burdens (*p*=0.0069) compared with those who report that their religion influences their opinion. There is no significant difference between responses of those who have and have not had previous education in LGBT+health disparities. There is no significant difference between male and female students' responses.

### Postlecture survey

**FIG. 2.**
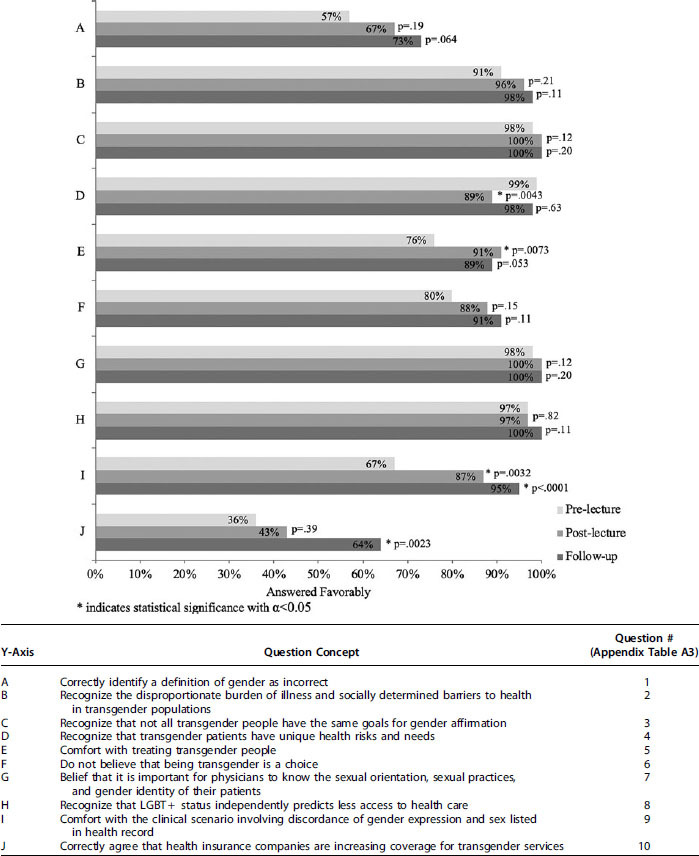
Proportion of favorable or correct responses and chi-square comparison of postlecture and follow-up surveys to the prelecture survey.

Seventy students completed the immediate postlecture survey (70% response rate) (Fig. 2). The proportion of students who were comfortable caring for transgender people changed from 65 (76%) to 64 (91%) (*p*=0.0073). The proportion of students comfortable with the described patient scenario significantly changed from 58 (67%) to 61 (87%) (*p*=0.0032). The proportion of students who were aware that transgender people have unique health risks and health needs significantly decreased from 85 (99%) to 62 (89%) (*p*=0.0043). LGB students, students with close LGBT friends, and students from urban environments (who answered the prelecture survey more favorably) represented a larger proportion of those who responded to the postlecture survey compared with the prelecture survey.

### One-year follow-up survey

Forty-four students completed the 1-year follow-up survey (44% response rate) (Fig. 2). Forty-two (95%) students were comfortable with the described patient scenario, differing significantly from the proportion of students who were comfortable with the scenario prelecture, which was 58 (67%) (*p*<0.0001). The proportion of students who were aware that insurance companies are increasing coverage for transgender health care significantly changed at 28 (64%) compared with 31 (36%) prelecture (*p*=0.0023). LGB students, students with close LGBT friends, and students from urban environments (who answered the prelecture survey more favorably) represented a larger proportion of those who responded to the follow-up survey compared with the postlecture survey.

## Discussion

In a group of medical students at two campuses, a 1-h lecture increased the proportion of students who could demonstrate accurate knowledge and favorable attitudes toward transgender patients immediately postlecture and up to a year after. Previous studies of transgender patient care training have shown retention up to 6 months, so longevity into subsequent training years could not be assumed before this study.^26^ The 1-year retention of the proportion of more accepting attitudes toward transgender people was impressive to observe after implementing only this first piece of the training, but not surprising given the ability of knowledge to impact attitudes and comfort level.^19^

The prelecture survey provides an important understanding of the student body. Only 25 (29%) of the student respondents had previous exposure to LGBT+ health disparity education before medical school. However, these students did not demonstrate more favorable attitudes or more knowledge regarding transgender people. This demonstrates the importance of this training for all future medical professionals, since prior training cannot be assumed to have taken place or be adequate. Another trend shown in prior literature but not these students was more accepting attitudes in female students compared with male students.^29^ These students could be different than those previously studied, but the survey was not designed to definitively test this.

Student groups that showed more favorable attitudes and more knowledge on transgender people included students who identified as LGBT+, those who have had close LGBT+ friends, students not from suburban environments, students who reported no influence of their religion on their attitudes or beliefs, and older students. Students from suburban environments were less likely to have had close LGBT+ friends compared with students from rural and urban environments, and so, it is possible that the environment of origin may influence attitudes and knowledge only to the extent that it is a proxy for having close LGBT+ friends. The superior impact of real-life experience with LGBT+ people over health disparity education supports the previously studied importance of a medical work force that values inclusivity and diversity.^24,30,31^ To leverage the value of interpersonal connections to foster understanding and acceptance, additional curriculum elements at the MCASOM will include transgender person encounters, a known high-impact learning modality.^32,33^

The postlecture survey showed a significant increase in the proportion of students who were comfortable caring for transgender patients (*p*=0.0073) and with the patient scenario (*p*=0.0032), which suggests that the lecture was successful for up to a year after. Interestingly, a significant decrease was seen in student knowledge that transgender people have unique health risks and health needs (*p*=0.0043). This could be explained by the fact that the lecturers emphasized that transgender people should be treated with the same respect and accommodation as other patients. Additionally, the session was brief and took place before the students received training on the particular medical needs of transgender patients. Therefore, it is possible that students misinterpreted this to mean that transgender people do not have unique health risks and needs. As a result, future trainings will teach medical and surgical aspects of transgender people's care, an often neglected part of training.^34–36^

The 1-year follow-up results show persistent improvement in the proportion of favorable attitude and knowledge. Before this study, the potential for learning benefits of a 1-h lecture on transgender patient care beyond 1 and 3 months was unknown.^26,27^ As far as the authors are aware, this is the first demonstration of the ability of the impact of a single lecture on transgender patient care to persist into subsequent training years. Therefore, this lecture can be effectively built upon throughout a longitudinal curriculum on caring for transgender people.

Since the training methods that generated the evidence on this topic lack standardization, the merits of this method of developing a training program on this topic warrant discussion. This incremental method of adding and studying each new training session as it is implemented has four key benefits. First, it enforces the development of longitudinal training for effective attitude change and knowledge retention.^7^ Second, it provides the opportunity for ongoing quality improvement and assurance. Third, it reduces the time and resources needed to deliver effective training by enabling faculty to know when learning objectives have been met. Fourth, it reduces the disruption that new trainings can cause to preexisting training elements. Herein lies a challenge. School administrators who adopt this methodology must recognize the necessity of continuing training development over a long period of time.

Previously published studies on this topic report a wide variety of teaching modalities, resource intensiveness, quality assessment techniques, and result validity.^23–27^ Relative effectiveness and cost/benefit ratios cannot be determined. Issues with external validity and logistical feasibility further undermine the usefulness of these studies for other institutions. Indeed, lack of curriculum development and improvement persists as a barrier to training implementation.^37,38^ The incremental approach described here may address this barrier through an individualized approach that builds in responsiveness to student needs, opportunity for quality assurance, and responsible use of the time and resources available to each institution.

### Limitations

It is possible that students who chose not to answer the postlecture survey more heavily represented the students who answered questions unfavorably in the prelecture survey. Nonatheistic (religious, including “prefer not to say”) students decreased from 67 (78%) before lecture to 52 (74%) after the lecture, and was 34 (77%) at 1 year. In addition, students who reported no close friendships with LGBT+ people decreased from 27 (31%) before the lecture to 16 (23%) after the lecture and 6 (14%) at 1 year. The same number of nonheterosexual students who answered the prelecture survey also answered the postlecture survey (*n*=9), with only one less nonheterosexual student response at 1 year. It is therefore possible that the positive change observed in answer percentages may not be representative of all students. As the survey was anonymous, pre- and postlecture surveys could not be compared directly with a paired *t*-test, significantly increasing the chance of type 2 error. The survey does not show which students were more receptive to the lecture material.

The diversity of factors with an impact on attitudes (having close LGBT+ friends, sexual orientation, environment growing up, religiosity, etc.) and small student body prevented stratification by these high-impact factors. In addition, anonymity may not fully protect against students' fear that the demographic information collected might be used to make generalizations about groups with whom students identify. Demographic information was collected at the end of the survey to lessen this influence.

The postlecture survey was collected before students received more medical training that could impact their responses. However, the results of the 1 year follow-up survey are subject to this confounding. The authors worked with the MCASOM Equity Curriculum Committee to map all curriculum components that could impact attitudes and knowledge on transgender people. Given the prolonged implementation process, additional exposure to concepts directly covered in the survey is minimal. Future studies on this curriculum will include analysis to measure the impact of subsequent training sessions.

### Strengths

Based on the variety between previously published curricula and preferential neglect for transgender health training, it is prudent to begin with a simple first intervention with special attention to attitudes toward transgender people that could be thoroughly studied over time. The logistical feasibility standards of all previous studies are conservatively maintained by use of a single subject-area expert delivering a 1-h lecture covering basic health disparity and terminology knowledge with focus on addressing bias.

This design protects against the limitations of internal validity observed in previous studies. Required attendance for all students further limited positive selection bias that would be present if students could self-select to attend the lecture. The assessment survey is brief and measures both knowledge and attitudes; and the high response rate immediately after the lecture further limits sampling bias.

The study design supports the generalizability of these result findings to other schools. Results show significant benefits of the lecture over two different student bodies, two different instructors, and two different geographic regions. Student body characteristics support generalizability of these results to other student populations and may be referenced to assess this (Table 1).

This study is effective in elucidating the learning needs of the student body, in improving the quality of the lecture, in demonstrating 1-year retention of increased proportions of favorable attitudes and accurate knowledge, and in serving as a first step in transgender patient care curriculum development at the MCASOM.

## Conclusion

A 1-h didactic session on transgender health improved the proportion of medical students with favorable attitudes and knowledge for at least a year. Students who are or have close personal experience with LGBT+ people have more favorable attitudes and correct knowledge regarding transgender people, supporting the importance of a diverse medical workforce and learning through contact with this minority group. Evaluation of the lecture enables improvement of the lecture and development of a multimodal, longitudinal, high-value curriculum tailored to the needs of the MCASOM students and availability of educational resources beyond the Mayo Clinic. The use of this methodology may reduce barriers to designing and implementing training, thereby narrowing the health outcomes gap of gender and sexual orientation minority patients.

## Ethical Approval

Institutional review board approval was granted for all aspects of this project. ID No. 17-005680.

## Previous Presentations

Poster presentation: A.J.N., C.J.D.-P., J.M.K., T.B.N., R.L.I. An Evidence-Based Approach to Education on Caring for Transgender Patients. ENDO, Chicago, IL. March 2018. Endocrine Reviews, Volume 39, Issue 2 Supplement, April 2018.

## Abbreviations Used

AAMC: Association of American Medical Colleges
LGBT: lesbian, gay, bisexual, and transgender
MCASOM: Mayo Clinic Alix School of Medicine

## Appendix

**Appendix Table A1.** The Mayo Clinic Alix School of Medicine Lecture Format Was Designed According to Educational Best-Practices

Content Chosen to satisfy learning objective guidelines^A1^ Address bias to encourage openness to subsequent sessions^A2^ Use resources that facilitate self-representation of minority groups^A3^ Learner expectations Clear learning objectives shared with students at beginning of lecture^A4^ Explicit definition of the lecture as a safe and affirming learning space^A1^ Modality Discussion for participatory learning^A5^ Multimedia to engage learners^A6^ Quality assessment and improvement Assessment guided by learning objectives^A7^ Follow previously determined protocol^A8^ Prompt feedback solicitation from students and teachers^A9^

**Appendix Table A2.** The Mayo Clinic Alix School of Medicine Lecture Content Was Chosen to Satisfy Association of American Medical Colleges-Recommended Learning Objectives, and to Advance Key Attitudes and Skills

MCASOM lecture content	AAMC-recommended learning objectives^A1^	Teaching to improve skills and attitudes
Explain the natural variety in sex, gender, gender expression, sexual orientation, and sexual practice. Discuss examples of people who are diverse in these characteristics.	Understand that sex, gender identity, gender expression, sexual orientation, and sexual practice exist on a spectrum and in any combination.	Attitude: lessen heteronormativity bias
Discuss and distribute list of common terms, with discussion of recommended language.	Recognize terminology that transgender people may find affirming or offensive.	Skill: improved ability to use affirming language
Discuss major illness burdens to transgender people.	Recognize the disproportionate burden of many health conditions in the transgender population.	Attitude: compassion for the vulnerable position occupied by transgender people in a discriminatory society Attitude: desire to not contribute to discrimination Attitude: desire to provide equitable care to transgender people
Discuss the minority stress that transgender people face.	Recognize the prevalence and impact of discrimination against transgender people.	
View and discuss video vignette demonstrating transgender discrimination in the health care setting.^A10^	Be aware of factors that create a hostile health care environment and approaches that can be used to create an inclusive health care environment.	
Discuss examples of starting conversations by asking for preferred pronouns, of avoiding stereotypes, and recovering from mis-gendering.	Understand methods of interacting with transgender people in an affirming way.	Skill: improved ability to have affirming patient interactions
Discuss and distribute list of resources for antidiscrimination legal services, advocacy groups, counseling services, inclusion of LGBT+ students and staff.	Identify local resources available to transgender youth and adults.	Skill: improved ability to connect transgender patients, staff, and students with resources

AAMC, Association of American Medical Colleges; LGBT+, lesbian, gay, bisexual, and transgender; MCASOM, Mayo Clinic Alix School of Medicine.

**Appendix Table A3.** Survey

Question no.	Question stem	Answer choices
1	Gender is a person's sense of being male, female, neither, or both, or along a spectrum. In addition, it describes who the person is attracted to.	True
		False
2	Transgender people have similar rates of mental health disorders/emotional stress, substance use disorders, and experience of physical violence/injury compared with the general population.	True
		False
3	Do all transgender people have the same goals for transition, for example, hormonal and surgical therapy?	True
		False
4	Transgender patients have unique health risks and health needs.	True
		False
5	How comfortable would you be with treating a transgender person?	Very comfortable
		Somewhat comfortable
		Somewhat uncomfortable
		Very uncomfortable
6	Do you believe that being transgender is a choice?	Yes
		No
7	Do you believe that, as a physician, it is important to know your patients' sexual orientation, sexual practices, and gender identity?	Yes
		No
8	Sexual minorities have the same level of access to health care services as individuals with similar socioeconomic status and race who are not sexual minorities.	True
		False
9	You have an appointment with a patient whose gender is listed as male in their medical record. The patient's first name is one that is stereotypically male. When you arrive in the examination room to meet the patient, they appear to be dressed in stereotypically female clothing and present as female. How comfortable would you feel initiating a discussion regarding their preferred pronouns and gender identity?	Very comfortable
		Somewhat comfortable
		Somewhat uncomfortable
		Very uncomfortable
10	Health insurance companies are increasing coverage for transgender services.	True
		False
11	Select the answer that best applies to you:	I have had close friends who are LGBT+.
		I have NOT had close friends who are LGBT+.
12	Please specify your gender:	Female
		Male
		Nonbinary/third gender
		Prefer to self-describe
		Prefer not to say
13	Please specify you sex assigned at birth:	Male
		Female
		Intersex
		Prefer not to say
14	Please specify your sexual orientation:	Gay/lesbian/homosexual
		Straight/heterosexual
		Bisexual
		Prefer to self-describe
		Prefer not to say
15	Please specify your ethnicity:	Hispanic or Latino or Spanish origin
		Non-Hispanic or Latino or Spanish origin
		Prefer not to say
16	Please specify your race:	Native American or Alaskan Native
		Asian
		Black or African American
		Native Hawaiian or other Pacific Islander
		White
		Prefer not to say
17	Please specify your age:	20–24
		25–29
		30–34
		35–39
		40–44
		Prefer not to say
18	Please specify your religion:	Atheist
		Christian
		Hindu
		Jewish
		Muslim
		Other
		Prefer not to say
19	How much does your religion (spirituality) impact your opinion on sexual practices, sexual orientation, family values, gender, and reproductive issues?	Not at all
		Somewhat
		Extremely
		Prefer not to say
20	Please specify the environment you grew up in:	Rural
		Urban
		Suburban
		Prefer not to say

### Appendix References

A1. Joint AAMC-GSA and AAMC-OSR Recommendations Regarding Institutional Programs and Educational Activities to Address the Needs of Gay, Lesbian, Bisexual and Transgender (GLBT) Students and Patients. 2007. Washington, DC: Association of American Medical Colleges. A2. Phelan SM, Burke SE, Hardeman RR, et al. Medical school factors associated with changes in implicit and explicit bias against gay and lesbian people among 3492 graduating medical students. J Gen Intern Med. 2017;32:1193–1201. A3. Greene RE, Hanley K, Cook TE, et al. Meeting the primary care needs of transgender patients through simulation. J Grad Med Educ. 2017;9:380–381. A4. Braun HM, Garcia-Grossman IR, Quiñones-Rivera A, et al. Outcome and impact evaluation of a transgender health course for health profession students. LGBT Health. 2017;4:55–61. A5. Noonan EJ, Sawning S, Combs R, et al. Engaging the transgender community to improve medical education and prioritize healthcare initiatives. Teach Learn Med. 2018;30:119–132. A6. Bonvicini K. LGBT healthcare disparities: what progress have we made? Patient Educ Couns. 2017;100:2357–2361. A7. Cannon SM, Shukla V, Vanderbilt AA. Addressing the healthcare needs of older lesbian, gay, bisexual, and transgender patients in medical school curricula: a call to action. Med Educ Online. 2017;22:1320933. A8. Committee on Lesbian, Gay, Bisexual, and Transgender Health Issues and Research Gaps and Opportunities. *Board on the Health of Select Populations. The Health of Lesbian Gay, Bisexual and Transgender People: Building a Foundation for Better Understanding.* Washington, DC: The National Academies Press, 2011. A9. McNair R. Outing lesbian health in medical education. Women Health. 2003;37:89–103. A10. National LGBT Cancer Network. Vanessa Goes to the Doctor, in LGBT Cultural Competency Curriculum. 2015. Available at www.lgbtcultcomp.org Accessed June 25, 2017.
